# Sertoli-Leydig cell tumor with unique nail findings in a post-menopausal woman: a case report and literature review

**DOI:** 10.1186/s13048-014-0083-5

**Published:** 2014-08-28

**Authors:** Dalia Moghazy, Chakradhari Sharan, Malika Nair, Cassandra Rackauskas, Robert Burnette, Michael Diamond, Omar Al-Hendy, Ayman Al-Hendy

**Affiliations:** Center for Women Health Research, Meharry Medical College, 1005 Dr. D.B. Todd Jr. Blvd., Nashville, Tennessee 37208 USA; Department of Pathology, Meharry Medical College, Nashville, Tennessee USA; Department of Obstetrics and Gynecology, Georgia Regents University, Augusta, Georgia

**Keywords:** Sertoli-Leydig cell tumor, Hirsutism, Virilization, Postmenopausal woman, Androgenic nail changes

## Abstract

**Background:**

Sertoli-Leydig cell tumor (SLCT) is a rare sex-cord tumor that usually occurs unilaterally and accounts for < 0.5% of all ovarian tumors. SLCT is uncommon in post-menopausal women, with the average age of diagnosis being 25 years.

**Case:**

We present a case of a 63-year-old post-menopausal woman presenting with progressive hirsutism, and male-pattern baldness. Unusual nail changes were also observed.

**Methods:**

Hormonal profile of the patient revealed increased testosterone and estradiol levels, and a 3.5 cm left ovarian mass. The patient was evaluated and was not found to be anemic or iron-deficient. Intraoperative frozen section assessment during laparoscopic exploration revealed SLCT, which was confirmed subsequently by histopathological and immunohistochemical (IHC) examination. Nail bed tissues were collected from normal females and evaluated by IHC for the presence of androgen receptors (AR).

**Results:**

The patient had an excellent postoperative course and all her testosterone-related manifestations were reversed within one year of surgery. Following surgery, the patient’s unique nail abnormalities also resolved gradually. The IHC evaluation also confirmed the presence of AR in nail bed tissues of females.

**Conclusion:**

SLCT, albeit rare, should be considered in post-menopausal women presenting with virilization and elevated androgen levels. Unusual nail signs may develop in response to increased androgen levels in these patients.

## Background

Sertoli-Leydig cell tumor (SLCT) is a rare sex cord-stromal tumor that accounts for less than 0.5% of all ovarian neoplasms [1]. Ovarian sex cord-stromal tumors develop from specific gonadal stroma that supports germ cell development and hormone secretion [2]. Approximately 75% of these tumors occur during reproductive age in the second and third decades of life, and less than 10% occur prior to menarche or after menopause [3]. Elevated levels of testosterone and androstenedione can be seen in approximately 80% of patients with ovarian SLCTs. As with other types of ovarian tumors, the patient can present with pain or abdominal distention due to the presence of a space-occupying lesion [3]. However, in contrast to other types ovarian tumors (epithelial or germ-cell), SLCTs may present with clinical signs and symptoms related to excess androgen production such as virilization [4]. Such manifestations include: progressive hirsutism, deepening of voice, male pattern scalp hair recession, increased musculature, and clitoromegaly. Patients can also present with estrogenic manifestations [5].

## Case

**Figure 1:**
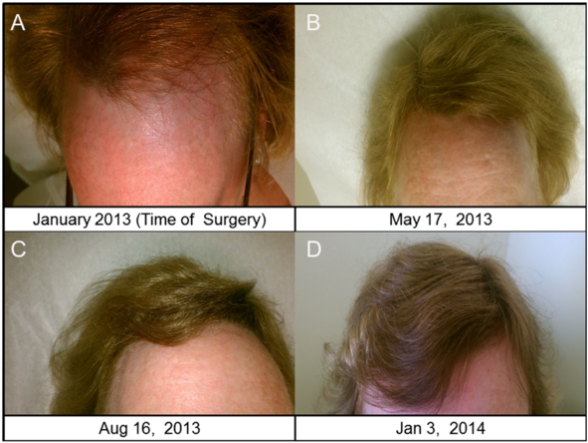
**Hairline changes. A)** Time of surgery hairline (receded) **B)** Hairline by 5 months post-surgery **C)** Hairline improvement by 8 months post-surgery **D)** Hairline improvement by 12 months post- surgery.

**Figure 2:**
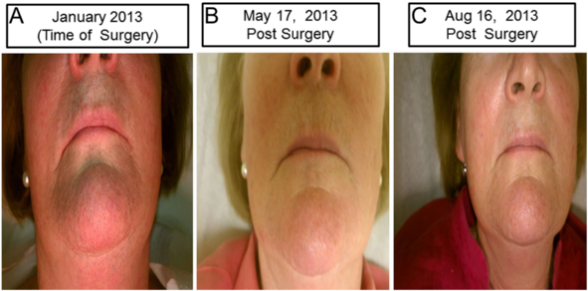
**Facial hair changes. A)** Time of Surgery - visible hair stubs **B)** Five months post- surgery **C)** Eight months post-surgery.

**Figure 3:**
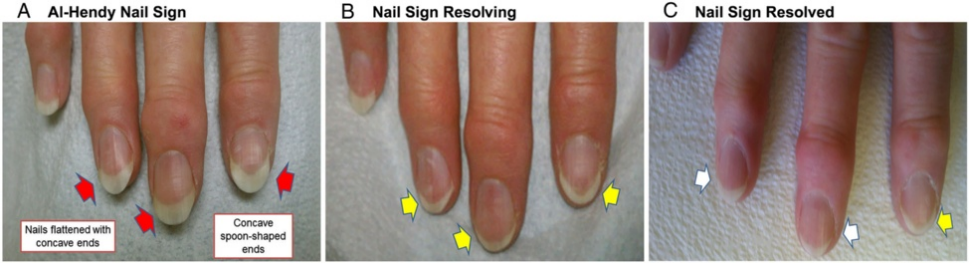
**Unique nail signs. A)** Al-Hendy Nail Sign (Red Arrows) Nails appear flattened with concave spoon shaped ends even 5 months post-surgery. **B)** Nail sign resolving (Yellow Arrows) by 8 months post-surgery **C)** Nail Sign resolved (White Arrows) on most fingers by one year post-surgery.

We present the case of a 63-year-old post-menopausal G3P3003 Caucasian female presenting with complaints of progressively increased hair growth, deepening voice, and a receding hairline over the past year. She also complained of some pelvic discomfort that resembled menstrual cramps, but denied overt vaginal bleeding. Her past medical history was significant for osteoporosis diagnosed twelve years prior. She denied any history of ovarian or adrenal disease. She denied any use of medications. The patient’s age of menarche was at 12 years and menopause at the age of 50 years. The patient’s last Pap smear was a year ago which was normal and she also reported no history of abnormal pap smears. The patient reported three normal pregnancies with uneventful cesarean sections and three living healthy children. Surgical history was remarkable for tonsillectomy and rhinoplasty. She was a happily married homemaker and exercised regularly. Interestingly, she noted an increased exercise tolerance and reported that she could easily double her normal biking routine. She also reported an increased libido. She denied tobacco, alcohol, or intravenous drug use. Family history was significant for hypertension in her mother and cardiovascular disease in her father. The subject provided written informed consent for us to use un-identifying photos of her affected areas. Physical exam was notable for marked facial stubble subsequent to shaving (Figure 1) and diffuse terminal hair across her chest and abdomen. There was also some regression in the patient’s hairline (Figure 2). There was no acanthosis nigricans present. Physical exam also revealed unique changes (Figure 3) in 8 of her fingernails (described below), however the toenails were normal. Cliteromegaly measuring 2 cm was observed on pelvic exam. The remainder of the physical exam was otherwise unremarkable. Laboratory studies showed an extremely high total testosterone and androstenedione level at 742 ng/dl (reference range: < 62 ng/dL) and 339.0 ng/dL (20-75 ng/dL) respectively. Serum DHEA sulfate 174.2 mcg/dL (<15 - 157 mcg/dL), TSH 1.6 μIU/mL (0.5 - 4.70 μIU/mL), calcium 9.9 mg/dL (8.8 - 10.3 mg/dL), and parathyroid hormone levels 44.2 pg/ml (11 - 54 pg/ml) were all within normal reference range. The serum estradiol level was slightly elevated at 54 pg/mL (0 to 30 pg/mL; postmenopausal) (Table 1). A computed tomography (CT) scan of the abdomen and pelvis showed a 3.5 cm left ovarian mass with normal appearing adrenal glands. This was subsequently confirmed by ultrasound which revealed an enlarged hypoechoic left ovarian mass of 3.6 × 3.5× 3.6 cm, and normal right ovary measuring 1.9 × 2.1 × 1.7 cm. Her uterus was normal in size, measuring 5.8 × 2.6 × 4.8 cm. But, the endometrial lining was heterogeneous and thickened, measuring at 9 mm. Preoperative and intraoperative consultation with our gynecological oncology team was conducted. The patient was referred for surgical removal of the ovarian mass. Laparoscopic bilateral salpingo-oophorectomy, peritoneal washings, and dilation and curettage were performed. At the time of surgery, ovaries, fallopian tubes, and uterine cutterings were sent for intra-operative frozen section pathology consultation. The frozen section of the left ovary revealed features of a “sex cord stromal tumor”. The right ovary was atrophic measuring 1.9 × 2.1 × 1.7 cm. The frozen section analysis of uterine curettings revealed no signs of atypia or malignancy. Lymph node dissection was not warranted based on preoperative and intraoperative evaluation as well as frozen section histological consultation showing a well-contained lesion within the ovary.

**Table 1:** **Serum parameter: pre- and post-surgery**

**Serum parameter (reference range)**	**1** ^**st**^ **visit**	**1 hour prior to surgery**	**1 week post surgery**	**5 months post- surgery**	**8 months post surgery**	**1 year post surgery**
**Total testosterone** (< 62 ng/dL)	742				<20	<20
**Free testosterone** (0.8 - 10 ng/dL)		455 ng/dL	<10 ng/dL	<10 ng/dL		
**Anderostendione** (<2 ng/ml)		3.390	0.285	0.259	0.474	0.319
**Progesterone **(< 1.0 ng/mL)		0.97	0.44	0.37		
**Estradiol** (0 to 30 pg/mL, postmenopausal)		54	<12	<12	<12	<12
**FSH** (25.8 - 134.8 mIU/ml, postmenopausal)		47.9	95.1	106.9	95	99.4
**LH **(14.2- to 52.3 mIU/mL, post-menopausal)		16.3	33.8	40.9	41.8	38.6
**DHEA-SO4** (<15-157 μg/dL)	174.2	102	21.1	24.5	24.3	36.1
**Ca** (8.8 - 10.3 mg/dL)	9.9					
**TSH** (0.5 - 4.70 μIU/mL)	1.6					
**Hgb **(12.1 to 15.1 g/dL, female)	13.1				16.1	
**Ferritin** (12-150 ng/mL, female)					65.1	

### Tumor pathology and immunohistochemical analysis

**Figure 4:**
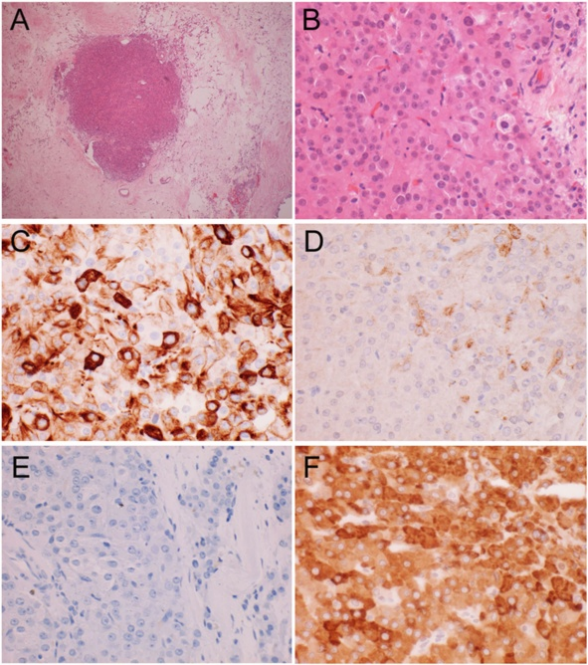
**Immunohistochemical staining of SRLC tumor. A)** Tumor nodule, (4×) **B)** Tumor section H&E stained (40×) Leydig cells and scattered Sertoli cells. **C)** Strong to weak CAM 5.2 staining 40× **D)** Weak and specific Keratin A/E staining 40× **E)** Epithelial Membrane Antigen (EMA) Negative. Sertoli and Leydig cells are both EMA negative, 40× **F)** Inhibin Positive, 40×. Both Sertoli and Leydig cells typically show a Calretinin+, CD99+, Inhibin + phenotype.

The final pathology report revealed a left ovarian Sertoli-Leydig cell tumor measuring 2.5× 2.0×1.4 cm. The tumor was composed primarily of Leydig cells, without well-formed Sertoli cell tubules or cord (Figure 4A). High-power (40×) fields revealed primarily sheets and aggregates of Leydig cells in a dense fibrous stroma (Figure 4B). Sertoli cells were difficult to identify. Immuno-histochemical stains for Keratin 8/18 or CAM 5.2 staining was mixed, strong to weak (Figure 4C). Both Keratin AE1/AE3 (Figure 4D) and Wilms tumor gene(WT-1) stains revealed negative to weak staining of Leydig cells and were positive for those considered as Sertoli cells. Negative staining of all cells for Epithelial Membrane Antigen (EMA) (Figure 4E) and diffuse, positive staining of all the cells for CD99, Calretinin, and alpha Inhibin, (Figure 4F) was observed, consistent with the pattern for both Sertoli cells and Leydig cells. Peritoneal washings were negative for tumor cells. The patient had a normal postoperative course and was discharged from the hospital on the first postoperative day in good condition.

The patient returned for a series of postoperative follow up visits. The first was one week post-surgery and then every three months for a total of one full year.

### Nail changes associated to hyperandrogenemia

Interestingly, careful exam of this patient pre-operatively revealed unique fingernail changes. The patient stated that she observed these changes months prior to presentation in the clinic. The nails looked mostly flat with significant curved concave spoon shaped ends. This was observed in most (8/10) fingers. The changes were particularly impressive in the index and middle fingers on both hands (Figure 3). An extensive review of the literature did not reveal similar findings in SLCT cases reported before nor any other female endocrinopathy. It is possible that previous cases of SLCT-induced hyperandrogenemia may have overlooked these nail findings. The only condition that leads to similar findings is iron-deficiency anemia. Although patient hemoglobin levels were always within the normal reference range, detailed iron-deficiency anemia work up was performed (Table 1) and was all within normal limits. Importantly, the fingernail changes started to gradually reverse after the curative surgery and normalization of patient hormonal profile (Table 1). The post-surgery reversal of nail changes (Figure 3) led us to confidently conclude that the nail signs were secondary to hyperandrogenemia. As this was the first association between fingernail changes and hyperandrogenemia, we propose these changes to be named the “Al-Hendy” nail sign.

### Expression of androgen receptor in nail bed area

Since androgens mediate all their cellular biological effects through its androgen receptor (AR), a member of the nuclear steroid receptor superfamily [6], we assumed that the fingernail area must express AR. We then decided to examine this concept ourselves. After appropriate IRB approval from Meharry Medical College, we collected nail bed tissues from healthy female volunteers and processed them for AR immunohistochemistry as described in Materials and Methods Section.

## Materials and Methods

The study was designed to evaluate the presence of androgen receptors in healthy female nail-bed cells. In this study we examined 5 healthy women between the ages of 20 to 50 years old. Inclusion criteria were: not currently pregnant or breastfeeding and greater than 6 months post-partum, post-abortion, post-pregnancy, or post-lactation at the time of collection. Participants must not have had any significant medical history or have taken any medications for three months prior to specimen collection. After informed consent was obtained, the participants were instructed to clip their own fingernails adjacent tissues at home and keep them in a 10% formalin solution that was given to them. Participants scheduled a time to drop off these tissues with the study coordinator. The nail bed tissues were processed for androgen receptor (AR) immunohistochemistry using standard protocols.

Tissues were briefly dehydrated and embedded in paraffin. The blocks were sectioned at a thickness of 5 μm for the immunohistochemical staining; the primary AR antibody was used at 1: 5, dilution. After washing with DPBS, a biotinylated secondary antibody (diluted 1: 200), was used for 10 min, following streptavidin-conjugated peroxidase for 15 min. The sections were stained with diaminobenzidine (DAB) substrate and counterstained using hematoxylin. Immunohistochemical (IHC) stains are important and useful in the pathological diagnosis of Sertoli- Leydig cell tumors, as the various cell types may be difficult to recognize on H&E (Hemotoxin and Eosin) stains alone. The IHC stains not only help verify the type of the tumor cells that are present, but also help to differentiate them from other tumor types that may share similar histologic features. For example, they can differentiate between tumors with malignant behavior (i.e. carcinoma) from the benign behavior of the Sertoli-Leydig cell tumor. Sertoli cells typically are cytokeratin+, and EMA-, whereas Leydig cells are both cytokeratin- and EMA-. Both cell types show a Calretinin+, CD99+, Inhibin + phenotype.

## Results

Post-op lab results revealed a swift and dramatic reduction in testosterone and estradiol to almost undetectable levels (Table 1). During the one year postoperative follow up, the patient indicated that her pelvic cramps discomfort had resolved. Physical exams confirmed that hirsutism elsewhere on her body had completely resolved, and that her scalp hair was regrowing (Figure 1B, C and D). She reported that she shaved her face less frequently and by the end of the year, she stopped shaving all together except infrequently shaving the chin area (Figure 2B and C). Her fingernails signs were resolving and returning to normal (Figure 3). She also noted that her hot flushes had come back. However, the hot flushes gradually subsided over time. The patient also noticed decreased libido and decreased exercise tolerance compared to the year prior to surgery. The patient continues to be on an annual follow up schedule.

**Figure 5:**
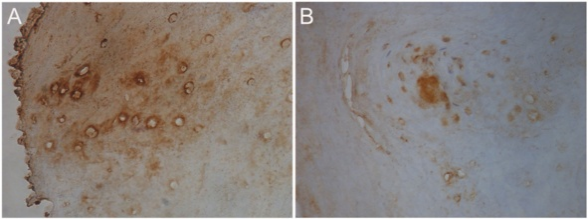
**Immunohistochemical staining of nail beds for androgen receptors. A)** Nail bed areas reveal strong AR staining (in all tested samples). **B)** Langerhans nests area of epidermis showing strong AR staining.

Following our novel observation of nail changes with the hyperandrogenemia in a post menopausal SLCT patient, we evaluated the presence of androgen receptors (AR) in the nail bed of normal healthy women. As shown in Figure 5, immunohistochemical analysis of nail bed areas revealed strong AR staining in all tested samples, especially in the Langerhans nests area of the epidermis (Figure 5A and B).

## Discussion

Most Sertoli-leydig cell tumors are unilateral and occur between the ages of 25-30, with an average age of diagnosis at 25 years. Around 50% of these cases come to clinical attention due to the progressive masculinization as seen in our patient. In a post-menopausal female, mild hirsutism is generally idiopathic. However, in a patient with increased levels of androgens and virilization, a work-up should be done to rule out an androgen-producing tumor of the ovaries or adrenal glands. An ovarian origin of the virilization can be suspected in the presence of elevated androgens with normal DHEA or a negative dexamethasone suppression test.

Sertoli and leydig cells are incapable of synthesizing estradiol [7] which suggests that the excess estradiol is due to aromatization of some of the abundantly available testosterone in this patient. As our patient was about 13 years postmenopausal with likely scant reservoir of follicular (granulosa) cells which are the main source of aromatase in reproductive age women [8], this is likely a peripheral aromatization process. Interestingly, this increased estrogen levels correlated very well with the patient’s complaint of menstrual-like pelvic discomfort although there was no overt uterine bleeding. A prior case report of SLCT by Guo et. al., 2012) [5] revealed postmenopausal bleeding and endometrial hyperplasia in an SLCT case in China. Although preoperative transvaginal ultrasound revealed a thickened endometrial lining of 9 mm, the endometrial curettings demonstrated no cancer, hyperplasia or atypia. This could be due to early intervention in our case that preempted prolonged exposure of the endometrium to persistently elevated levels of endogenous unopposed estradiol or other individual modifiers. Importantly, concurrently with the dramatic postoperative drop in androgen level, the estrogen level returned to normal postmenopausal level. Again, this confirms that androgen-aromatization was the source of excess estradiol in the patient. The patient’s menstrual-like pelvic discomfort quickly resolved postoperatively which is likely due to the removal of the estradiol stimulation of endometrium. FSH and LH levels increased after surgery as the level of estrogen decreased (Table 1) which suggests that ovarian-hypothamic-pituitary axis feedback is still operational many years post-menopause, as suggested in previous reports [9]. This patient described the return of hot flushes after surgery, which she described as her second menopause. This was likely due to estrogen-withdrawal after the oophorectomy procedure and consistent with the well-established notion that hot flushes are due to estrogen withdrawal [10]. The patient was hesitant to use hormone replacement therapy to treat hot flushes and instead the patient successfully used exercise to manage this particular complaint [11].

The patient’s unique nail changes pre-surgery (Al-Hendy nail sign) and the subsequent reversal in nail curvature post-surgery reveals that androgens might have a direct effect on human nails and nail bed tissues. Other causes of spooning include iron deficiency anemia and hemochromatosis. However, we found the patient’s CBC and ferritin to be within normal reference range. The conclusion that androgens are very likely the causative agent behind these unique nail changes are based on the observation that these changes started to resolve after the normalization of serum androgen levels postoperatively in this patient. Similar to other steroids, androgens act on target cells by diffusing through the plasma membrane and binding to specific intracellular/nuclear receptors [6]. Since androgens induce biological effects only by signaling via androgen receptors (AR), we presumed that there are androgen receptors located within nail bed tissues. Samples of the female nail bed showed strong positive AR staining concentrated in the Langerhans area. This is the first report of AR in human nail bed area. We intentionally used premenopausal donors for the nail bed androgen expression studies as androgen receptor is an estrogen-regulated gene and thus would be affected by the menopausal status and will not reflect our patient’s preoperative hormonal milieu which featured severe hyper-androgenemia and mild hyper-estrogenemia. Immunohistochemistry rather than in vitro studies culturing tissue on the freshly fixed samples was used to avoid potential changes in androgen receptor. The concentrations of AR in the Langerhans cells, which act as antigen-presenting cells [12], suggest that androgens might play a role in immune function of skin. Interestingly, we could not detect AR expression in male nail bed samples that we obtained from several men and concurrently tested (data not shown). An effect of androgens on digits in women should not have been totally unexpected. It is well known that a sexual dimorphism identified to be related to prenatal androgen level exists in the ratios of the index finger to the ring finger (2D:4D ratio) [13,14]. In males, the 2D:4D ratio is smaller than in females, and prenatal androgen exposure of female fetuses reduces the 2D:4D ratio.

Ideally, we would have liked to study the expression of androgen receptor in the index case herself. Several limitations prevented us from accomplishing that. First, the patient was not easily accessible due to an extensive travel schedule. Additionally, due to the novelty of the observation, we did not know for sure if the nail changes were due to androgen excess until the changes began to resolve after the surgical removal of the SLCT tumor. Androgen, like other steroid hormones, induce its own receptor and hence measuring AR levels after surgery would not have reflected the status before surgery during the severe hyper-androgenemia phase. Future studies will address these limitations.

## Conclusion

Sertoli-Leydig cell tumors are a rare ovarian sex-cord tumor that usually occurs unilaterally. The diagnosis should always be considered in a young female that presents with symptoms of progressive virilization and ovarian mass on examination. However, albeit rare, this diagnosis should also be considered in post-menopausal females with similar symptoms. Detailed imaging studies should be promptly performed to search for the malignancy. Management of ovarian SLCT remains challenging due to lack of standard management protocol. Fortunately, since the vast majority of SLCTs are unilateral and confined to the ovaries in 90% of cases, surgical resection is generally curative. Prognosis is related to the tumor staging. The overall 5-year survival rate for stage I is 95% whereas stages III and IV is nearly 0%. Long-term follow-up is advised in all patients. This case revealed a unique and novel finding of nail changes associated with hyperandrogenism which demonstrates that androgen receptors can be found in the female nail bed. The clear connection of specific finger nail changes (Al-Hendy nail sign) and hyperandrogenemia awaits further confirmation from similar cases to reveal if this is limited to postmenopausal hyperandrogenemia or if it is also evident in reproductive age women. If confirmed to be present widely, this nail sign which is easy to detect, might function as a simple non-invasive screening tool for easy and early detection of hyperandrogenic endocrinopathies in women. Future studies looking at the effects of androgens on nails are warranted.

## Consent

All patient information used in the manuscript were de-identified and anonymized. A written consent was obtained from patient to use un-identifying photos of affected areas in this publications. Formal IRB consent was collected from subjects donating tissues for the androgen receptor studies.

## Abbreviations

SLCT: Sertoli-Leydig cell tumor
IHC: immunohistochemical
EMA: Epithelial membrane antigen
WT-1: Wilms tumor gene
AR: Androgen receptor
DAB: Diaminobenzidine

## References

[1] Young RH, Dudley AG, Scully RE (1984). Granulosa cell Sertoli - Leydig cell and unclassified sex cord-stromal tumors associated with pregnancy: a clinicopathological analysis of thirty-six cases. Gynecologic oncology.

[2] Schultz KP, Frazier L, Schneider DT, Frazier AL, Amatruda JF (2014). Ovarian and Testicular Sex Cord-Stromal Tumors. Pediatric Germ Cell Tumors.

[3] Roth LM, Anderson MC, Govan AD, Langley FA, Gowing NF, Woodcock AS (1981). Sertoli - Leydig cell tumors a clinicopathologic study of 34 cases. Cancer.

[4] Osborn RH, Yannone ME (1971). Plasma androgens in the normal and androgenic female: A review. Obstetrical & Gynecological Survey.

[5] Guo L, Yang X, Zhu H, Qiu W, Shi X, Huang B, Duan T (2012). Sertoli-Leydig cell tumor presenting hyperestrogenism in a postmenopausal woman: a case report and review of the literature. Taiwanese journal of obstetrics & gynecology.

[6] Meehan KL, Sadar MD (2003). Androgens and androgen receptor in prostate and ovarian malignancies. Frontiers in bioscience: a journal and virtual library.

[7] Speroff L, Fritz M (2005). Clinical gynecologic endocrinology and infertility.

[8] Garzo VG, Dorrington JH (1984). Aromatase activity in human granulosa cells during follicular development and the modulation by follicle-stimulating hormone and insulin. American journal of obstetrics and gynecology.

[9] Downs JL, Wise PM (2009). The role of the brain in female reproductive aging. Molecular and cellular endocrinology.

[10] Freedman RR (2001). Physiology of hot flashes. American Journal of Human Biology.

[11] Hammar M, Berg G, Lindgren R (1990). Does physical exercise influence the frequency of postmenopausal hot flushes?. Acta Obstet Gynecol Scand.

[12] Murakami R, Denda-Nagai K, Hashimoto SI, Nagai S, Hattori M, Irimura T (2013). A unique dermal dendritic cell subset that skews the immune response toward Th2. PloS one.

[13] Berenbaum SA, Bryk KK, Nowak N, Quigley CA, Moffat S (2009). Fingers as a marker of prenatal androgen exposure. Endocrinology.

[14] Nowak NT, Mofatt SD (2011). The relationship between second and fourth digit ration, spatial cognition, and virtual navigation. Archives of Sexual Behavior.

